# Snakebite outbreak and associated risk factors in Donga, Taraba State, Nigeria, June, 2016

**DOI:** 10.11604/pamj.2020.37.82.17288

**Published:** 2020-09-22

**Authors:** Philip Bobu Igawe, Jibreel Omar Muhammad, Ugochukwu Uzoechina Nwokoro, Joshua Difa Abubakar, Salisu Idris Isah, Udi Aketemo, Muhammad Shakir Balogun, Patrick Nguku

**Affiliations:** 1 Nigeria Field Epidemiology and Laboratory Training Program, Abuja, Nigeria,; 2 Ministry of Health, Jalingo, Taraba State, Nigeria

**Keywords:** Snakebite, risk factors, Nigeria, *Echis spp*

## Abstract

Snakebite envenoming is a public health problem among rural communities in Nigeria. In June, 2016, an outbreak of snakebites in Donga Local Government Area, Taraba State, north-east Nigeria, was reported. We investigated the outbreak to identify risk factors for snakebites and to institute appropriate control measures. We conducted an unmatched case control study to identify risk factors for snakebite in the communities involved. We conducted key informant interviews and Focus Group Discussions with stakeholders in the communities to obtain information on the community´s perspective of the outbreak. There were Sixty-one (61) snakebite cases with Fifteen (15) deaths [CFR 24.6%]. Majority of the mortalities [37(60.3%)] were males. Median age was 27 years (Range: 5-58). Kadarko ward had the highest [26 (42.6%)] number of cases. Most snakebites 12 (44.4%) occurred in the farm, 27 (96.4%) vipers Echis spp were responsible for most of the bite and most [26 (92.9%)] victims sought care from traditional healers. Residing in Kadarko ward and having a history of snakebite in the past were risk factors [Odds ratio of 2.9 (95% CI 1.1-7.4) and 5.9 (95% CI 1.1-32.5)] respectively. Abandonment of homes for two years due to communal clashes has been thought to have allowed snake populations to grow. The snakebite outbreak in Donga, Taraba State affected predominantly male farmers in the rural wards. Residing in Kadarko ward and having a previous history of snakebite were risk factors.

## Introduction

Rural populations are frequent victims of snakebites as they go about their daily crop production and animal rearing activities and as they reside in their homes. Snakebite is a neglected public health problem [1]. Clinicians have for a long time witnessed the tragedy of injury, disability, and death from snakebite that is a daily occurrence in many parts of Africa, Asia, and Latin America. To many people living in these regions, including some of the world's poorest communities, snakebite is an ever-present occupational risk and environmental hazard, an additional penalty of poverty. Like malaria, dengue, tuberculosis, and parasitic diseases, the risk of snakebite is always present. Unlike many of these other public health risks, however, the burden of human suffering caused by snakebite remains largely unrecognized. The problem is so underrated that it was only added to WHO's list of neglected tropical diseases in April, 2009. Yet an estimated 5.4-5.5 million people are bitten by snakes each year, resulting in about 400 000 amputations, and between 20 000 and 125 000 deaths globally [2]. The mortality due to snakebites fluctuates between 1 and 22% [3]. The population most at risk being mostly young men engaged in agricultural or pastoral labors [4].

In Nigeria snakebite envenoming is a major public health problem among rural communities of the savanna region. The Saw-scaled or Carpet viper *Echis ocellatus* and, to a lesser extent, the African cobras *Naja spp*. and puff adders *Bitis arietans* have proven to be the most important cause of mortality and morbidity [5]. Taraba State is located in North-Eastern Nigeria with the capital in Jalingo. It has sixteen Local Government Areas (LGA) with a projected population of 3,035,153 based on the 2006 census. It occupies a land area of 54,473 Km square, lies roughly between latitude 6030^3^ and 9036^3^ north and longitude 90 10^3^ 50^3^ east. It is bounded by Bauchi and Gombe States in the north-west and Adamawa on the north-east, by Plateau State in the west. The state is further bounded to the south-west by both Nasarawa and Benue States, while it shares an international boundary with the Republic of Cameroun to the south and south-east.

The region lies largely within the tropical zone and vegetation of low forest in the southern part with mountainous features and grassland in the northern part. Rivers Benue, Donga, Taraba and Ibi are the main rivers in the state [6]. Donga LGA has the dry and rainy season common to tropical regions. The rainy season starts in April and ends in October, while the dry season begins in November and terminates in March. The climatic, soil and hydrology of the State provide a conducive atmosphere for the cultivation of most staple food crops, grazing land for animals, and freshwater for fishing as well as forestry. The vegetation comprises the Guinea Savannah which is marked by mainly forest and tall grasses are found in the southern part of the State like Wukari, Ussa, Kurmi, Takum and Donga, the natural topography of the region is mostly forested with varying degrees of thick and sparse vegetation, it has rocky hills and mountains that favors the breeding of reptiles and other fauna [6]. Species of snake reported to be common in the state include; carpet vipers, puff adders and cobras. Cases of snakebite commonly involving carpet vipers are usually reported during rainy season in the southern part of the state.

A national daily reported that *No fewer than 27 persons have been confirmed dead in Donga Local Government Council of Taraba State following the various degrees of injuries they sustained from snakebites ravaging rural agrarian communities in the Local government area*. On the first of June 2016 [7]. The State Disease Surveillance and Notification system recorded an even higher number of snakebite cases, the LGA has not recorded such high number of cases and mortalities from snakebites. The outbreak of snakebite in Donga LGA was declared by the state government on the 6^th^of June. The Nigeria Field Epidemiology and Laboratory Training Programme (NFELTP) deployed residents to the state to (I) confirm the existence of the outbreak, (ii) characterize the outbreak in terms of the persons involved, the places and time of occurrence of cases, (iii) Identify the predominant snake specie involved in driving the high number of cases and deaths. (iv) identify the risk factors and (v) provide technical support for the control of the outbreak.

## Methods

The team conducted active case search in the various communities in all the wards affected by the snakebite outbreak in Donga Local Government Area. The wards visited during the active case search were Kadarko, Akate, Mararraba, Suntai, Fada, Nyita, Garanya and Asibiti wards. We defined as a case, as any person residing in Donga LGA reported to have been bitten by a snake from the 17th March, 2016. A control was a person residing in the same community or village with the case, with no report of snakebite during the same period.

**Study design:** an unmatched case-control study was conducted.

**Sample size:** using 95% CI, power of 80%, OR of 4, frequency of exposure among controls of 20% and case to control ratio of 1: 2, a total sample size of 84 respondents (28 cases and 56 controls) was calculated using Epiinfo version 7. software.

**Study population:** persons of any age living in the affected communities in Donga LGA, Taraba State with snakebite (cases) or without snakebite (controls). Two types and sources of data were collected, we obtained the line list of cases from the office of the LGA´s Disease Surveillance and Notification Officer (DSNO) and updated same after active case search. We collected information regarding demographic characteristics and potential risk factors through interviewing participants using an adapted standardized semi-structured interviewer-administered questionnaire.

**Data processing and analysis method:** data obtained from the questionnaires was entered into a computer database using Epiinfo version 7. software. Line listed data were entered into a Microsoft excel sheet. The data was summarized using descriptive and analytic statistics. MS Excel was used to generate tables, graphs and charts. We compared cases with controls through the calculation of odds ratio and 95% confidence intervals. We conducted all statistical analysis using Epiinfo version 7. software.

## Results

Results of Descriptive studies revealed that there was a total of 61 snakebite cases with 15 deaths, with a case fatality rate (CFR) 24.6%. the most affected sex group were males 37 (60.3%). The age range of cases was 5 to 58 years with a median age of 27 years. People aged 15 to 44 years accounted for 45 (73.8%) cases. Cases were reported in 8 of the 10 wards in Donga LGA. Kadarko ward reported the highest number of cases with 26 (42.6%) cases followed by Akate ward with 12 (19.7%) cases, Asibiti ward with 9 (14.8%) cases and Suntai ward with 7 (11.5%) cases (Table 1). Kadarko ward was observed to have the highest number of cases (42.6%) while the least (1.6%) number of snakebite cases was reported in Fada and Garaya wards with a case each. Suntai ward had the highest CFR (42.9%) and no mortalities were observed in Mararaba, Nyita, Fada and Garaya wards. There were 61 cases of snakebite, fifteen mortalities and a 24.6% CFR (Table 1).

**Table 1:** distribution of snakebite cases by wards, Donga LGA, Taraba State, June, 2016

Wards	Cases (%)	Deaths	Ward specific CFR%
Kadarko	26 (42.6)	5	19.2
Akate	12 (19.7)	4	33.3
Asibiti	9 (14.8)	3	33.3
Suntai	7 (11.5)	3	42.9
Mararaba	3 (4.9)	0	0
Nyita	2 (3.3)	0	0
Fada	1 (1.6)	0	0
Garaya	1 (1.6)	0	0
Total	61	15	24.6

**Figure 1:**
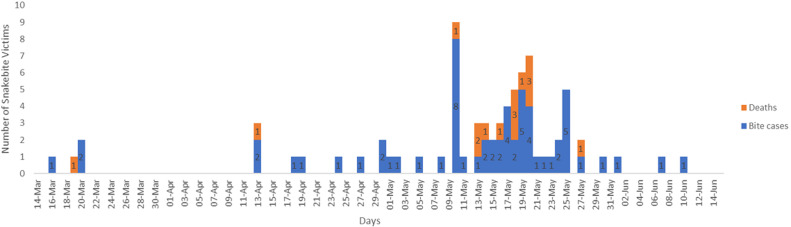
epidemic curve of snakebite outbreak, Donga LGA, Taraba State, March to June, 2016

The epidemic curve (Figure 1) illustrates that the outbreak started on 17^th^ March, 2016 with a bimodal peak on the 10^th^ and 20^th^ May, 2016. Most mortalities were recorded within the 9^th^ to the 28^th^ of May 2016. Most snakebite cases 12(44.4%) occurred in the farm or around farming activities. Most victims of snakebites 26(92.9%) sought care from a traditional healer as the first point of care, and a high percentage (35.7%) of cases of snakebite were not reported to the health authorities. hence there were official confirmed hospital records and verbal reports of snakebite occurrences. In most cases 27(96.4%) the viper was identified *Echis spp* responsible for the bite (Table 2). A total of 84 respondents (28 cases and 56 controls) were recruited in the study of unmatched case control study (1 case to 2 controls) to identify risk factors for snakebite in Donga LGA, Taraba State. People with previous snakebite history were observed to be at significant risk of being a victim of snakebite during the outbreak, (OR 5.9) CI 1.1-32.5. Residing in Kadarko ward was also found to have the odds [2.9(1.1-7.4)] of being a case (Table 3).

**Table 2:** patterns and characteristics of snakebite occurrence

Variables	Frequency	Percentage (%)
**Where did you seek First Aid**		
Clinic/hospital	2	7.1
Traditional Healer	26	92.9
**Type of snake involved**		
Viper	27	96.4
Cobra	1	3.6
Other	0	0
**Reported to Health authorities**		
Yes	18	64.3
No	10	35.7
**Site of Bite wound**		
Upper Limb	9	32.2
Lower Limb	17	60.7
Others	2	7.1
**Place Bite occurred**		
Farm	12	44.4
Home	10	35.7
Others	6	21.4

Frequencies and percentages from the case control study

**Table 3:** association between snakebite and identified risk factors, snakebite outbreak, Donga LGA, Taraba State, March to June, 2016

Variables	Cases (n=28)	Controls (n=56)	Odds Ratio(95%CI)
**Age**			
<18	3	2	3.2 (0.5 - 20.6)
≥18	25	54	
**Sex**			
Female	11	22	1.0 (0.4 - 2.5)
Male	17	34	
**Occupation**			
Farming	22	39	1.6 (0.5 - 4.6)
Others	6	17	
**Level of Education**			
No formal education	10	12	2.0 (0.7 - 5.6)
Formal education	18	44	
**Ward of residence**			
Kadarko	15	16	2.9 (1.1 - 7.4) *
Others	13	40	
**Previous history of snakebite**			
Yes	5	2	5.9 (1.1 -32.5) *
No	23	54	
**Past history of snakebite among household member**			
Yes	17	27	1.7 (0.7 - 4.2)
No	11	29	

* Significant association at 95% confidence limit

## Discussion

The investigation revealed that cases of snakebites were reported in Donga LGA from 17^th^ March 2016 with a bimodal peak on 10^th^ and 19^th^ May, 2016. The peak snakebite period corresponded with the commencement of the rainy season with most residents of Donga LGA returning to their communities to farm. This might have increased the man-reptile interface resulting in increase in snakebite episodes [8]. There was a total of 61 snakebite cases with 15 deaths, CFR = 24.6%. The high CFR observed in this study is similar to findings from other studies conducted in Kaltungo, Gombe State, north-east Nigeria prior to the use of the Echis-specific anti-snake venom and also in other parts of Africa [3, 9] but is much higher than the 1.41% reported after the use of Echitab® the Echis-specific anti-snake venom for treatment of snakebite cases there [10]. We believe that the CFR recorded in Donga LGA could have been significantly reduced to a very low level with the prompt use of anti-snake venom as has been demonstrated from the Kaltungo study mentioned above. Males (60.3%) were more affected, same has been reported in other studies in Africa and India [4, 11] as males are primarily the breadwinners in these communities and as such are more at risk of snakebites during farming activities or other pastoral endeavors.

Our study also noted that the active age group of 15 to 44 years was the most affected (%). This is similar to findings from studies in Nigeria and some other parts of Africa and India [4, 11]. Kadarko ward reported the majority of cases and also had the highest CFR. The reason for this finding is unclear. Most snakebite cases occurred in the farm or among those involved in agricultural and livestock activities. This supports the findings reported by other researchers where most cases of snakebite occurred among rural farmers [1, 2, 4]. Either the saw-scaled or carpet viper *Echis spp* (96.4%) was the predominant specie of snake involved in the snakebite outbreak in Donga LGA. This is similar to the findings of other researchers in north-east Nigeria and other parts of the country [5, 12]. This is bit partly because this is one of the most common type of snake in the region [5].

Our investigation also revealed that majority (92.9%) of the cases sought care at traditional healing homes, as their first or only place visited for medical treatment of the snakebite. This corresponds to findings from similar studies in north-east Nigeria and other parts of Africa where traditional healing practices and other superstitious beliefs are rampant [1]. In addition, our investigation revealed that most of the cases and other community members were unaware of the free anti-snake venom and other medications provided by the Taraba State government as the designated treatment centre (The first referral hospital in Donga, Donga LGA). While we did not find a statistically significant association between age, gender, occupation or level of education with being a case, we found that residing in Kadarko ward and having a history of snakebite in the past were statistically significantly associated with being a case of snakebite in the current outbreak with Odds ratio of 2.9 (95%CI 1.1-7.4) and 5.9 (95%CI 1.1-32.5) respectively. This implies that in our study, persons residing in Kadarko ward had almost three times the odds of being a case compared to those residing in other wards. Equally, those with a previous history of snakebite had almost six times the odds of being a case compared to those without a previous history of snakebite. Community members reported that most of the villagers have just recently returned to their homes after being away from their homes for almost two years following a communal clash. The absence of human activities may have allowed for the unhindered breeding of the snakes in the community.

**Limitations:** active case search was limited by insecurity in some of the affected communities due to inter-ethnic and farmers/herdsmen clashes making them inaccessible. As many cases patronized traditional healers rather than the designated treatment centre in Donga, we depended on the ward focal persons and the traditional healers to obtain our line list of cases. There might have been recall bias.

**Public health actions taken:** immediate sensitization of the community members and traditional healers to report cases and to use the government designated treatment centre. Health education of the community on the preventive measures against snakebite and other animal stings.

**Recommendations:** the following were recommended to the State Ministry of Health: increased community sensitization and education on preventive measures; continuous advocacy to community leaders especially on need for prompt reporting of cases and utilization of free medication and decentralize the treatment centre to include Primary Healthcare Centres PHCs/Private Health facilities that good storage facilities and competent personnel for benefit of communities far from the LGA headquarters.

## Conclusion

The snakebite outbreak in Donga LGA, Taraba State affected predominantly male farmers in the rural wards of the LGA and was mainly due to bite by either the saw-scaled or carpet viper *Echis spp*. residing in Kadarko ward and having a previous history of snakebite were risk factors. Prompt and effective use of Echis-specific anti-snake venom would help reduce mortality.

### What is known about this topic

- There is an outbreak of snakebites in Donga LGA, Taraba State, Nigeria;
- Rural agrarian communities are affected;
- People were dying from these snakebites.

### What this study adds

- Snakebites affected predominantly male farmers in the rural wards of Donga LGA;
- Most cases were due to bite by either the saw-scaled or carpet viper Echis spp;
- Residing in Kadarko ward of Donga LGA and having a previous history of snakebite were important risk factors.
